# Transcriptomic and proteomic profiling of *Actinobacillus pleuropneumoniae* responses to iron starvation

**DOI:** 10.3389/fcimb.2025.1669654

**Published:** 2025-09-25

**Authors:** Yifang Cui, Jiahe Chen, Xu Feng, Fangfang Guo, Yuxin Shao, Fuzhou Xu

**Affiliations:** ^1^ Beijing Key Laboratory for Prevention and Control of Infectious Diseases in Livestock and Poultry, Institute of Animal Husbandry and Veterinary Medicine, Beijing Academy of Agricultural and Forestry Sciences, Beijing, China; ^2^ College of Animal Science and Technology, Beijing University of Agriculture, Beijing, China; ^3^ College of Agriculture and Animal Husbandry, Qinghai University, Xining, China

**Keywords:** *Actinobacillus pleuropneumoniae*, transcriptome, proteome, iron starvation, virulence factor, adaptation

## Abstract

**Background:**

*Actinobacillus pleuropneumoniae* (APP) is the causative agent of porcine contagious pleuropneumonia, which remains a major pathogen endangering the swine industry. However, the mechanisms underlying its colonization and pathogenesis in pigs remain largely unknown.

**Methods:**

An integrated analysis combining transcriptomic and proteomic profiling was employed to detect genetic and protein changes in APP under iron starvation.

**Results:**

In total, 458 differentially expressed genes (DEGs) from the transcriptome and 532 differentially expressed proteins (DEPs) from the proteome were identified. The comparative analysis showed that 137 differentially expressed genes/proteins were shared between DEGs and DEPs, with the majority exhibiting consistent regulatory changes at both transcription and protein levels. Functional enrichment analysis revealed that the downregulated genes were predominantly associated with the generation of precursor metabolites and energy (45/105, 42.86%), primary metabolic process (29/105, 27.62%), ion binding (20/105, 19.05%), and metal cluster binding (18/105, 17.14%), corresponding to pathways involved in primary metabolites and energy biosynthesis and cellular respiration. Conversely, the upregulated genes were primarily enriched in iron transport (11/30, 36.67%) and iron binding (9/30, 30%), which corresponded to the iron starvation conditions. The expression changes of iron utilization systems, including TonB-ExbB-ExbD and some TonB-dependent receptors, by qRT-PCR were consistent with the results in both transcriptome and proteome analyses.

**Conclusion:**

This study provided a global perspective on the response mechanisms employed by APP to iron starvation, characterized by suppressing electron transport and energy metabolism pathways and upregulating the pathways associated with the TonB-ExbB-ExbD energy transduction system for iron acquisition.

## 1 Introduction

*Actinobacillus pleuropneumoniae* (APP) is the causative agent of porcine infectious pleuropneumonia (PCP), a highly contagious respiratory disease affecting swine (Sassu et al., 2018). Infection with APP not only causes acute high mortality in pigs but also persists in the tonsils and lungs of subclinical infected pigs, triggering potential epidemic outbreaks (de Almeida et al., 2025). APP is classified into 2 biotypes and 19 serovars (Stringer et al., 2021). However, the cross-immune protection among different serovars is weak, which poses great challenges for controlling PCP (Sassu et al., 2018). Current commercial vaccines and control measures offer limited protection against APP infection. Therefore, it is necessary to develop novel and efficient prevention and control strategies.

Iron is an essential element for bacteria, participating in diverse cellular processes including respiration, ATP generation, and DNA replication and repair (Cassat and Skaar, 2013; Kramer et al., 2020). Additionally, the iron acquisition of pathogenic bacteria modulates their pathogenicity (Spiga et al., 2023). The host employs nutritional immunity, sequestering free iron through iron-binding proteins (such as transferrin and lactoferrin), compelling APP to utilize efficient iron acquisition systems to maintain survival and pathogenicity (Frost and Drakesmith, 2025). Therefore, the iron acquisition system is generally recognized as a major virulence factor in pathogenic bacteria (Buettner et al., 2009). Several iron transport systems have been identified in APP, including a transferrin receptor complex (TbpA/TbpB), a hydroxamate siderophore receptor (FhuA), and a hemoglobin-binding receptor (HgbA) (Jacques, 2004). It has also been shown that APP can use exogenous siderophores and may secrete endogenous chelators in response to iron starvation (Jacques, 2004). Studies have shown that TbpB exhibits serovar specificity (serovars 1, 5, and 7 of APP), and antibodies against TbpB can block iron uptake in these serovars (del Río et al., 2005). Consequently, the iron transporters of APP represent promising targets for vaccine design.

Bacteria possess adaptive mechanisms to cope with changing environments, including nutrient limitations and various stresses. This adaptation involves extensive reprogramming of gene expression and the specific induction or repression of key genes (Moreno-Gámez, 2022). Omics is often used to explore the expression patterns of global genes in bacteria. Transcriptional profiling of APP under iron-restricted conditions has been reported (Nielsen and Boye, 2005; Deslandes et al., 2007; Klitgaard et al., 2010). However, single-transcriptome analysis fails to capture all possible post-transcriptional regulatory mechanisms and translational regulation. The combination of transcriptome and proteome can reveal the mechanisms across complementary molecular levels. In this study, we performed an integrated analysis of transcriptomic and proteomic data to comprehensively investigate the mechanisms employed by APP in response to iron starvation. These findings establish a foundation for further deciphering the colonization and pathogenic mechanisms of APP in pigs and provide new insights for the development of control strategies.

## 2 Materials and methods

### 2.1 Strain and growth conditions

*Actinobacillus pleuropneumoniae* serovar 1 reference strain 4074 (ATCC 27088) was routinely cultured in tryptic soy broth (TSB, Difco, Sparks, MD, USA) or on agar supplemented with 5 μg/mL of nicotinamide adenine dinucleotide (NAD, Merck KGaA, Darmstadt, Germany) at 37°C. According to the results of the pilot studies (**Supplementary Figure S1**), 20 μM of deferoxamine mesylate (DFO, Merck KGaA, Darmstadt, Germany) was added to the TSB medium to establish an iron-restricted condition, and the TSB medium without supplements was used as the control.

### 2.2 RNA sample preparation

To determine the expression of genes related to iron utilization in APP, the experiment was divided into two groups: APP cultured in TSB + NAD medium serving as the control group and APP cultured in TSB + NAD + DFO medium under iron-restricted conditions serving as the iron-restricted group. The specific operations are as follows: fresh cultivated APP cells were harvested to collect the bacterial cells. Cell pellets were resuspended and washed with sterile PBS. APP in the control group was resuspended in TSB + NAD medium (group control), and the iron-restricted group was resuspended in TSB + NAD + DFO medium (group DFO). Bacterial suspensions were adjusted to approximately 10^5^ CFU/mL. Cultures were incubated at 37°C with shaking at 200 rpm for 4 h. Finally, APP cells from each group were collected by centrifugation for RNA extraction. The total RNA was extracted using TRIzol™ reagent (Thermo Fisher Scientific Inc., USA) according to the manufacturer’s instructions. Only a high-quality RNA sample (RNA quality number ≥ 8.0) was used to construct the sequencing library.

### 2.3 Transcriptome sequencing and data processing

The collected RNA samples were subjected to RNA-seq transcriptome sequencing (Shanghai Majorbio Bio-pharm Technology Co., Ltd., Shanghai, China). The RNA-seq library was prepared using the Illumina Stranded mRNA Prep Ligation method and sequenced on the NovaSeq X Plus platform (PE150). High-quality clean reads were obtained and aligned to the reference genome (APP serovar 1 strain 4074, GenBank accession number CP029003.1) for sequence mapping and alignment analysis. Gene expression levels were quantified using the transcripts per million (TPM) reads method. The RSEM software (http://deweylab.github.io/RSEM/) was used to quantify gene abundance. Differential gene expression analysis was performed using the DESeq2 software (http://bioconductor.org/packages/stats/bioc/DESeq2/). Genes exhibiting a log2 fold change (log2FC) ≥1 and an adjusted *P*-value (FDR) <0.05 were considered to be differentially expressed genes (DEGs). In addition, the identified DEGs were subjected to the Gene Ontology database for functional annotation and enrichment analysis.

### 2.4 Protein sample preparation

Proteomic sample preparation followed transcriptomic protocols (Section 2.2). Total proteins from the control and DFO groups were dissolved in a protein lysate solution containing 8 M of urea, 1% SDS, and a protease inhibitor cocktail. After lysis, proteins were digested with trypsin. The resulting peptide fragments were reconstituted in 0.1% trifluoroacetic acid, desalted using an Oasis HLB column (Waters Corporation, Framingham, MA, USA), and quantified by NanoDrop™ One (Thermo Fisher Scientific Inc., USA) prior to mass spectrometry detection.

### 2.5 Proteome sequencing and data processing

The peptide samples from different treatment groups were analyzed by a Vanquish Neo system coupled with an Orbitrap Astral mass spectrometer (Thermo Fisher Scientific Inc., USA) via the Majorbio platform. The chromatography run time was set to 8 min. Data-independent acquisition (DIA) was performed using an Orbitrap Astral mass spectrometer operated in DIA mode. The mass spectrometry scanning range was 100–1,700 *m*/*z*. DIA raw data were processed and searched using the Spectronaut software (Biognosys Inc., Newton, MA, USA). Protein quantification was performed using the MaxLFQ algorithm.

The database used for proteomic data analysis was the proteome of *Actinobacillus pleuropneumoniae* in the UniProt database (https://www.uniprot.org/), supplemented with annotation results derived from our transcriptome sequencing. Analogous to the transcriptomic analysis, the proteomics data were analyzed by calculating the relative protein expression ratio between groups and the corresponding *P*-value from statistical tests. Fold change and *P*-values for the proteins between the two groups were calculated using the “*t*-test” function in R. Proteins meeting the thresholds of fold change (FC) >1.2 or FC <0.83 and *P*-value <0.05 were defined as differentially expressed proteins (DEPs). Functional annotation and pathway enrichment analysis were performed on all identified proteins using the GO database and the KEGG database. Protein-protein interaction (PPI) networks were constructed using the STRING software (http://string-db.org).

### 2.6 Integrative analysis of transcriptome and proteome

An integrative analysis of transcriptomic and proteomic data was conducted to identify key factors and elucidate molecular mechanisms underlying iron utilization in APP using the Majorbio Cloud platform (https://cloud.majorbio.com/) (Han et al., 2024). The DEG and DEP sets were cross-referenced to identify shared genes/proteins. A union set of all DEGs and DEPs was also generated. Genes related to iron utilization within the intersection and union sets were identified, and the correlation between mRNA and protein expression levels for these selected genes was assessed.

### 2.7 Validation of DEGs by quantitative reverse transcription PCR

The expression of selected upregulated DEGs associated with iron acquisition was validated by quantitative reverse transcription PCR (qRT-PCR). RNA samples, prepared identically to those used for transcriptome and proteome sequencing, were utilized for validation. The selected genes and primer information are listed in **Supplementary Table S1**. 16S rRNA served as the endogenous control (Li et al., 2024). The qRT-PCR assay was performed using the CFX96 Connect™ Real-Time PCR System (Bio-Rad Laboratories, Hercules, CA, USA). The thermal cycling parameters were initial denaturation at 95°C for 3 min, followed by 39 cycles of denaturation at 95°C for 10 s, and annealing at 60°C for 30 s. A melting curve analysis from 60°C to 95°C was conducted after the amplification reaction. Relative gene expression was calculated using the 2^−ΔΔCt^ method (Livak and Schmittgen, 2001).

### 2.8 Statistical analysis

Statistical analyses were performed using Student’s *t*-test and one-way ANOVA by GraphPad Prism 8 (San Diego, CA, USA). Data were presented as means ± standard deviations. A *P*-value <0.05 was considered statistically significant.

## 3 Results and discussion

### 3.1 Identification and functional enrichment analysis of DEGs from the transcriptome

**Figure 1:**
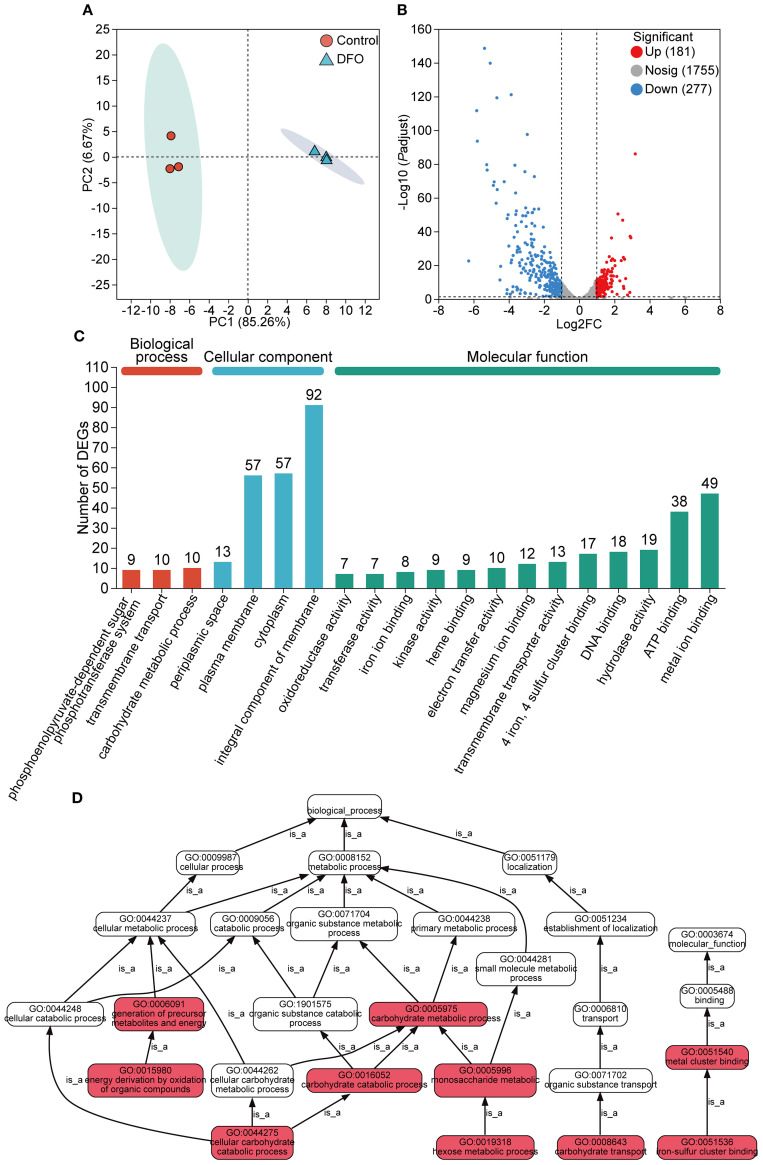
Gene expression analysis of the transcriptome in APP responding to iron starvation. **(A)** Principal component analysis of gene transcription profile of APP under iron-restricted conditions compared with the control group. **(B)** Differentially expressed genes (DEGs) with upregulation and downregulation between the iron-restricted and control groups. **(C)** Functional annotation of DEGs based on the GO database. The number of DEGs was labeled on the top of bars. **(D)** GO pathway enrichment analysis of DEGs. Red boxes indicated that DEGs were significantly enriched in the GO term. Each “is_a” link along the arrow direction represented the subclass relationship.

Firstly, principal component analysis (PCA) was performed to assess the overall distribution of the gene transcription profile. The iron-restricted and control groups with three independent biological replicates were clustered separately (**Figure 1A**), indicating high reproducibility within biological replicates and significant transcriptional differences between the two groups.

Gene expression levels between the iron-restricted and control samples were compared. A total of 458 DEGs were identified (**Supplementary Table S2**), comprising 437 annotated genes, 13 novel transcripts, and 8 small RNAs (sRNAs). Among these DEGs, 181 were significantly upregulated and 277 were significantly downregulated in response to iron starvation (**Figure 1B**).

All the DEGs underwent GO term annotation and enrichment analysis. The DEGs were annotated into three GO categories, including biological process (BP), cellular component (CC), and molecular function (MF), with significant enrichment observed for 29 terms in BP, 219 in CC, and 216 in MF (**Figure 1C**). Within the MF category, terms related to metal ion binding and transfer were significantly enriched. The top significantly enriched GO pathways for all 458 DEGs are shown in **Figure 1D**. Key enriched pathways included cellular metabolic processes, encompassing subcategories such as the establishment of localization and transport of cellular carbohydrate and organic substances.

### 3.2 Identification and functional enrichment analysis of DEPs from the proteome

**Figure 2:**
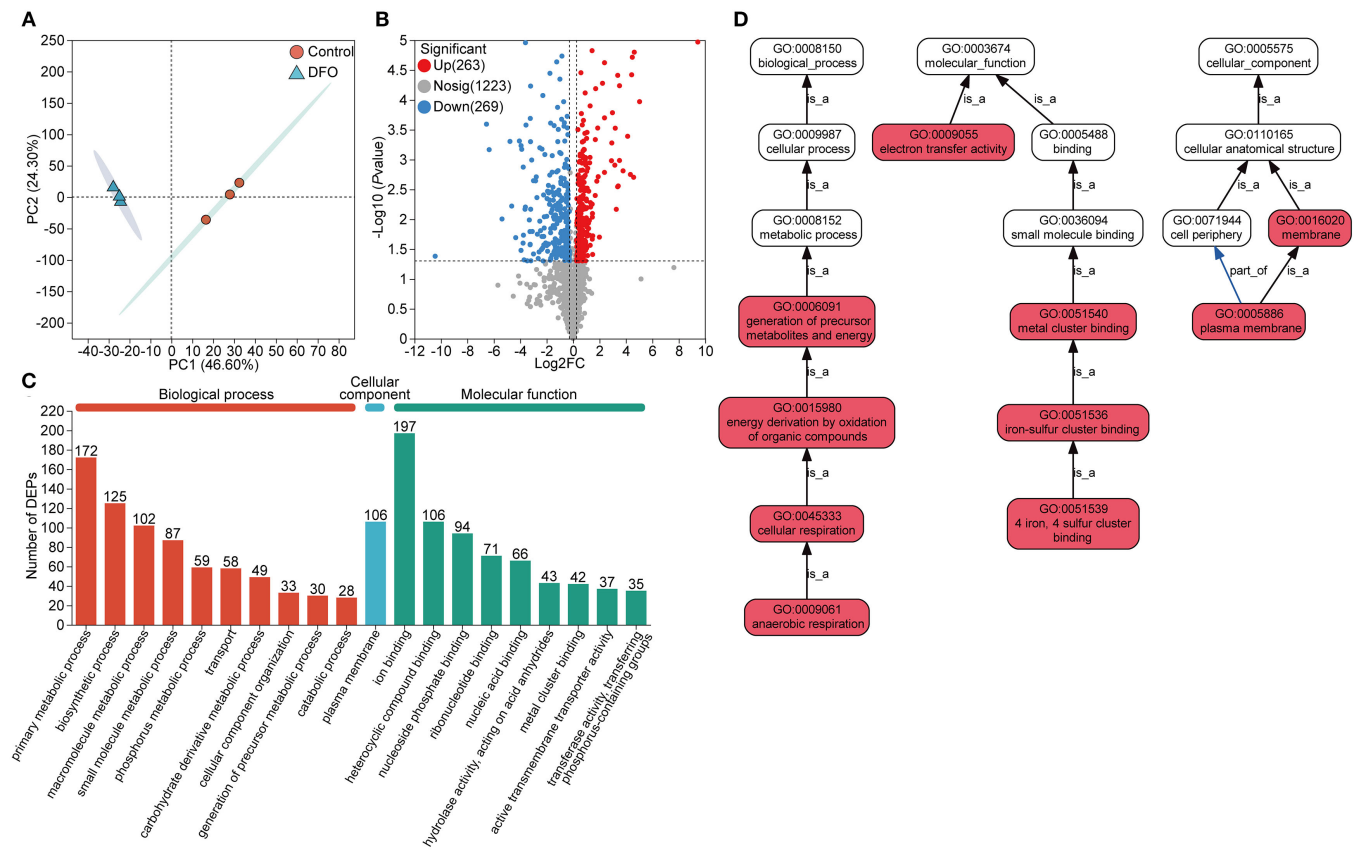
Protein expression analysis of the proteome in APP responding to iron starvation. **(A)** Principal component analysis of protein expression of APP under iron-restricted conditions compared with the control group. **(B)** Differentially expressed proteins (DEPs) with upregulation and downregulation between the iron-restricted and control groups. **(C)** Functional annotation of DEPs based on the GO database. The number of enriched proteins was labeled on the top of bars. **(D)** GO pathway enrichment analysis of DEPs. Red boxes indicated that DEPs were significantly enriched in the GO term. The “is_a” denoted subclass relationships and the “part_of” indicated inclusion relationships.

PCA analysis of the proteomic data also revealed substantial changes in the protein profile of APP under iron starvation compared to the control (**Figure 2A**). A total of 532 DEPs were identified (**Figure 2B**, **Supplementary Table S3**), with 263 upregulated DEPs and 269 downregulated DEPs. Similar to the transcriptomic analysis, DEPs were annotated into the three main GO categories: BP, CC, and MF. Among these, 197 DEPs were enriched in ion binding, which was the most dominant subcategory (**Figure 2C**). The DEPs were significantly enriched in pathways involved in core cellular processes, particularly energy metabolism, respiration, and molecular interactions such as iron-sulfur cluster binding and electron transfer (**Figure 2D**).

### 3.3 Integrated bioinformatic analysis of transcriptome and proteome

**Figure 3:**
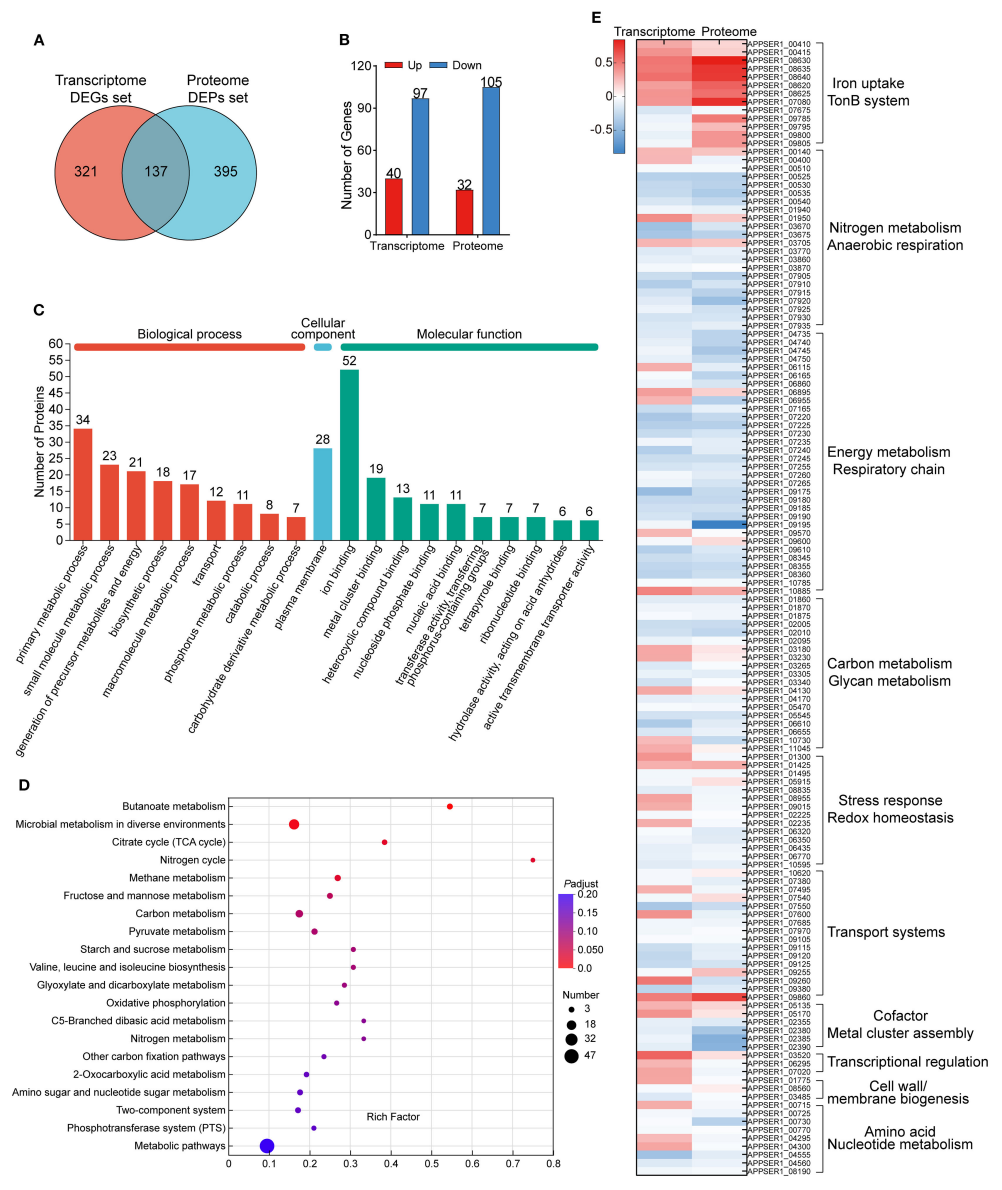
Shared genes and their encoding proteins in both the transcriptome and proteome. **(A)** Venn diagram of the number of DEGs in the transcriptome and DEPs in the proteome. **(B)** The expression profile of the shared 137 genes between the transcriptome and the proteome. **(C)** Functional annotation of the shared 137 genes based on the GO database. **(D)** KEGG pathway enrichment analysis of the shared 137 genes. **(E)** Heatmap of the expression of the shared 137 genes in APP under iron starvation in transcriptomic and proteomic sequencing. The scale bar represented the logarithm of normalized relative expression levels.

Integration of the 458 DEGs and 532 DEPs revealed that 137 genes and their corresponding proteins were shared between transcriptomic and proteomic analysis (**Figure 3A**). Among the 137 shared genes, 40 genes were significantly upregulated and 97 genes were significantly downregulated at the transcriptional level. Moreover, 32 proteins were significantly upregulated and 105 proteins were significantly downregulated referring to the proteome (**Figure 3B**). Furthermore, the up- or downregulated expression trends between the transcriptome and proteome were largely concordant for these genes. The fold change of upregulation was generally greater at the protein level compared to the transcriptional level. Discordant regulation between transcriptional and protein levels was observed for 28 genes (28/137, 20.44%, **Supplementary Table S4**).

The categories of the 137 genes were annotated through the GO database and enriched by the KEGG database (**Figures 3C, D**). Overall, the major annotated GO terms for the 137 shared genes included primary metabolic processes (34 genes), encompassing functions like generation of precursor metabolites and energy and oxidoreductase activity and ion binding (52 genes), including metal ion binding, metal cluster binding, and electron transfer. The expression levels of the shared 137 genes in APP under iron starvation in transcriptomic and proteomic sequencing are exhibited in **Figure 3E**.

In addition, the enrichment of two datasets of genes/proteins in the metabolic pathways of APP was analyzed. One dataset consisted of the shared 137 DEPs/DEGs as described above, and the other dataset included 395 DEPs only detected in the proteome (with no significant differences at the transcriptional level). As shown in **Supplementary Figure S2**, the shared DEGs/DEPs were mainly enriched in pathways, including carbohydrate metabolism (amino sugar and nucleotide sugar metabolism), terpenoid and polyketide metabolism (terpenoid backbone biosynthesis), energy metabolism (D-alanine metabolism, valine/leucine/isoleucine biosynthesis, TCA cycle), and the biosynthesis of other secondary metabolites (indole alkaloid biosynthesis, pantothenate and coenzyme A biosynthesis). Meanwhile, the DEPs only expressed at the protein level were mainly involved in lipid metabolism, carbohydrate metabolism (lipopolysaccharide biosynthesis, interconversion between pentose and glucuronate), nucleotide metabolism (purine and pyrimidine metabolism), and the biosynthesis of other secondary metabolites (metabolism of arginine, proline, valine, leucine, and isoleucine).

### 3.4 Downregulated genes under iron starvation

**Figure 4:**
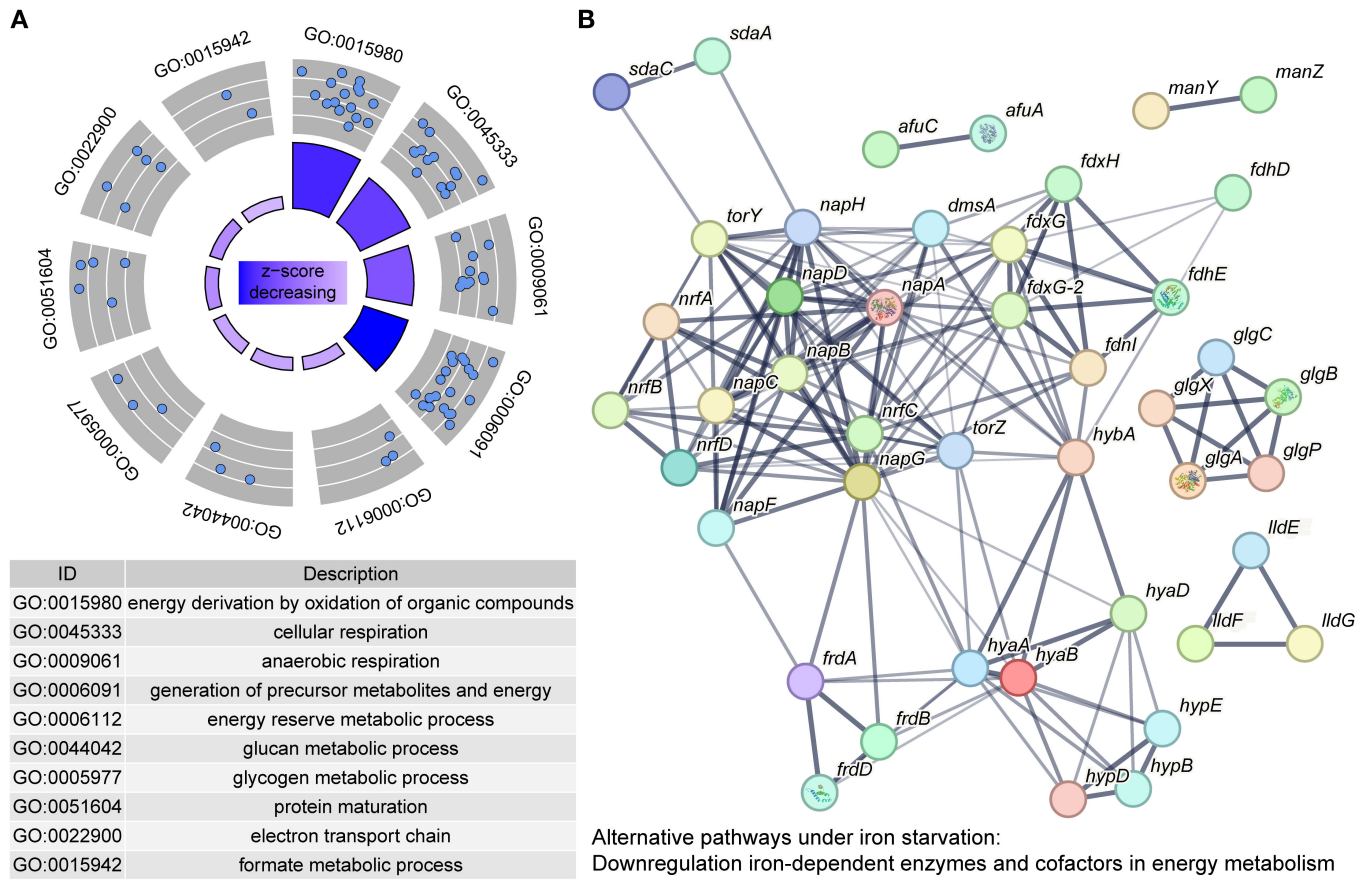
Downregulated genes in APP under iron starvation. **(A)** Enrichment of downregulated genes based on the GO database. Each blue dot represented a protein enriched in the GO term. The *z*-score in the inner circle was used to estimate the degree to which the pathway may be inhibited. **(B)** A protein interaction network was constructed by protein-protein interaction information from the STRING database. Each node in the network represents a distinct protein, and the thickness of the edges connecting the nodes indicates the strength of evidence for the protein-protein interaction.

The significantly downregulated DEGs (277/458, 60.48%) and DEPs (269/532, 50.56%) predominated in response to iron starvation, which revealed significant downregulation of iron-dependent pathways in APP, likely as an adaptation to conserve iron and mitigate an energy deficit. By cross-comparing the shared genes screened from transcriptomic and proteomic data, the proportions of downregulated genes were 70.80% (97/137) and 76.64% (105/137) in the transcriptome and proteome, respectively. Functional annotation and pathway enrichment analysis were performed on the 105 downregulated proteins identified in the proteome. The functional enrichment of these downregulated proteins is shown in **Figure 4A**. Within the BP category, the most enriched terms were “generation of precursor metabolites and energy” (45/105, 42.86%) and “primary metabolic process” (29/105, 27.62%). Within the MF category, “ion binding” (20/105, 19.05%) and “metal cluster binding” (18/105, 17.14%) were prominently enriched. Furthermore, pathway analysis revealed significant enrichment in primary metabolite and energy biosynthesis pathways, as well as cellular respiration (**Table 1**). The interaction network among these pathways is shown in **Figure 4B**. In agreement with previous reports (Deslandes et al., 2007; Klitgaard et al., 2010), APP primarily suppresses electron transport and energy metabolism pathways under iron starvation.

**Table 1:** Downregulated genes in the transcriptome and proteome of APP under iron starvation.

Gene operon	Gene	Accession ID^a^	Transcriptome		Proteome		Description
			Log2FC^b^	*P-*value	Log2FC	*P-*value	
*dms*	*dmsA*	APPSER1_09175	−6.27	1.03E−24	−3.09	1.60E−02	Dimethyl sulfoxide reductase subunit A
	*dmsB*	APPSER1_09180	−3.83	2.63E−25	−3.25	2.68E−02	Dimethylsulfoxide reductase subunit B
	*dmsC*	APPSER1_09185	−3.36	9.49E−22	−2.57	3.53E−02	Dimethyl sulfoxide reductase anchor subunit
	*dmsD*	APPSER1_09190	−2.90	5.17E−17	−3.25	2.63E−02	Tat proofreading chaperone DmsD
*man*	*manZ*	APPSER1_09115	−3.51	2.41E−31	−1.48	9.13E−03	PTS mannose transporter subunit IID
	*manY*	APPSER1_09120	−3.99	9.31E−34	−1.35	2.12E−02	PTS mannose/fructose/sorbose transporter subunit IIC
	*manX*	APPSER1_09125	−3.75	4.52E−28	−2.35	6.59E−04	PTS mannose transporter subunit IIAB
*frd*	*frdD*	APPSER1_08345	−3.49	1.97E−33	−2.46	1.55E−02	Fumarate reductase subunit D
	*frdC*	APPSER1_08350	−4.08	2.81E−50	–^c^	–	Fumarate reductase subunit FrdC
	*frdB*	APPSER1_08355	−4.03	1.47E−52	−3.13	1.01E−03	Succinate dehydrogenase/fumarate reductase iron-sulfur subunit
	*frdA*	APPSER1_08360	−4.26	1.83E−72	−2.79	1.94E−03	Fumarate reductase (quinol) flavoprotein subunit
*nap*	*napF*	APPSER1_07905	−3.50	2.42E−42	−3.76	1.85E−02	Ferredoxin-type protein NapF
	*napD*	APPSER1_07910	−4.83	2.44E−72	−2.27	5.00E−02	Reductase
	*napA*	APPSER1_07915	−3.03	8.54E−57	−3.78	2.51E−02	Periplasmic nitrate reductase subunit alpha
	*napG*	APPSER1_07920	−2.37	9.29E−22	−5.38	9.89E−03	Ferredoxin-type protein NapG
	*napH*	APPSER1_07925	−1.63	1.56E−11	−2.52	1.08E−02	Quinol dehydrogenase ferredoxin subunit NapH
	*napB*	APPSER1_07930	−2.94	1.21E−34	−2.22	1.94E−02	Nitrate reductase
	*napC*	APPSER1_07935	−2.64	5.42E−54	−1.44	3.52E−02	Cytochrome C
*afu*	*afuC*	APPSER1_07675	−2.71	5.11E−41	−0.65	3.98E−02	Ferric ABC transporter ATP-binding protein
	*afuB*	APPSER1_07670	–	–	–	–	Iron ABC transporter permease
	*afuA*	APPSER1_07665	−3.18	6.79E−20	−0.89	2.02E−01	ABC transporters
*hyp*	*hypB*	APPSER1_07220	−5.22	1.75E−79	−3.24	1.95E−04	Hydrogenase nickel incorporation protein HypB
	*hypD*	APPSER1_07225	−4.65	1.03E−67	−3.94	5.76E−04	Hydrogenase formation protein HypD
	*hypE*	APPSER1_07230	−3.69	4.52E−39	−2.23	3.91E−03	Hydrogenase expression/formation protein HypE
	*hypF*	APPSER1_07235	−1.48	1.23E−23	−1.53	6.16E−03	Carbamoyltransferase HypF
	*hyaA*	APPSER1_07240	−4.71	1.42E−59	−1.61	3.99E−02	Hydrogenase 2 small subunit
	*hybA*	APPSER1_07245	−3.60	1.01E−52	−2.84	2.27E−03	Hydrogenase 2 operon protein HybA
	*——*	APPSER1_07250	−2.57	1.27E−37			Ni/Fe-hydrogenase cytochrome b subunit
	*hyaB*	APPSER1_07255	−2.91	6.94E−54	−1.93	3.39E−03	Hydrogenase 2 large subunit
	*hyaD*	APPSER1_07260	−1.31	5.71E−09	−1.58	8.51E−04	HyaD/HybD family hydrogenase maturation endopeptidase
	*hybE*	APPSER1_07265	−1.60	4.41E−16	−2.29	7.46E−05	Hydrogenase-2 assembly chaperone
*fdo*	*fdhD*	APPSER1_04730	−0.22	2.65E−01			Formate dehydrogenase accessory sulfur transferase FdhD
	*fdoG*	APPSER1_04735	−2.45	1.86E−27	−3.64	6.79E−03	Sulfate ABC transporter substrate-binding protein
	*fdnG*	APPSER1_04740	−1.97	5.25E−25	−3.61	6.24E−03	Formate dehydrogenase-N subunit alpha
	*fdoH*	APPSER1_04745	−1.56	2.22E−14	−4.65	6.01E−03	Formate dehydrogenase subunit beta
	*fdoI*	APPSER1_04750	−1.90	3.85E−15	−3.31	1.03E−02	Formate dehydrogenase subunit gamma
	*fdhE*	APPSER1_04755	−0.56	7.68E−04	−1.25	1.60E−03	Formate dehydrogenase accessory protein FdhE
*sda*	*sdaC*	APPSER1_04555	−5.78	8.74E−97	−1.62	3.46E−03	Serine transporter
	*sdaA*	APPSER1_04560	−2.49	1.76E−28	−1.08	3.24E−03	L-serine ammonia-lyase
*tor*	*torZ*	APPSER1_03670	−5.82	5.23E−115	−2.19	1.40E−02	Trimethylamine-N-oxide reductase TorA
	*torY*	APPSER1_03675	−5.37	1.19E−152	−3.77	1.33E−03	Nitrate reductase
*lld*	*lldG*	APPSER1_02380	−2.23	9.16E−13	−4.78	4.87E−04	Lactate utilization protein C
	*lldF*	APPSER1_02385	−1.67	2.92E−07	−6.55	2.46E−04	Iron-sulfur cluster-binding protein
	*lldE*	APPSER1_02390	−2.17	4.03E−16	−6.35	6.73E−04	(Fe-S)-binding protein
*glg*	*glgB*	APPSER1_01855	−3.03	6.84E−23	−1.22	8.17E−02	1%2C4-alpha-glucan branching protein GlgB
	*glgX*	APPSER1_01860	−2.27	6.86E−21	−1.69	4.70E−02	Glycogen debranching enzyme GlgX
	*glgC*	APPSER1_01865	−2.70	3.59E−34	−1.70	8.87E−02	Glucose-1-phosphate adenylyltransferase
	*glgA*	APPSER1_01870	−1.56	2.01E−18	−0.96	3.89E−02	Glycogen synthase
	*glgP*	APPSER1_01875	−1.12	1.45E−08	−0.90	8.02E−03	Glycogen/starch/alpha-glucan phosphorylase
*nrf*	*nrfA*	APPSER1_00525	−4.50	4.13E−13	−3.94	3.32E−02	Ammonia-forming nitrite reductase cytochrome c552 subunit
	*nrfB*	APPSER1_00530	−3.86	1.45E−23	−3.71	4.80E−02	Cytochrome c nitrite reductase pentaheme subunit
	*nrfC*	APPSER1_00535	−3.75	6.45E−09	−4.34	2.03E−02	Cytochrome c nitrite reductase Fe-S protein
	*nrfD*	APPSER1_00540	−2.76	9.20E−24	−3.19	4.97E−02	Cytochrome c nitrite reductase subunit NrfD

a Accession ID in the genome of APP serovar 1 reference strain 4074 (CP029003.1).

b Mean of three independent samples; FC, fold change.

c No available data.

#### 3.4.1 Anaerobic respiratory chains

The anaerobic respiratory chains were coordinately downregulated (**Table 1**). The dimethyl sulfoxide reductase (*dms* operon), fumarate reductase (*frd* operon), periplasmic nitrate reductase *nap* locus, the TMAO reductase (*tor* operon), and cytochrome C nitrite reductase (*nrf* operon) were significantly downregulated (Baez et al., 2022). APP likely downregulated iron-dependent anaerobic respiration to conserve iron (essential for Fe-S clusters and heme cofactors) and reduce reliance on this energy-generating pathway (Lill and Freibert, 2020). In addition, APP inhibited hydrogen metabolism. The genes involved in [Ni-Fe]-hydrogenase maturation (*hyp* operon) and hydrogenase structural subunits (*hya*/*hyb*) were strongly downregulated, potentially eliminating an alternative energy source in response to iron limitation (Rios-Delgado et al., 2025).

#### 3.4.2 Energy metabolism pathways

The pathways involved in amino acid metabolism (*sda* and *lld*), glycogen metabolism (*glg*) and transport (*man*), and nitrogen metabolism (*nrf*) were downregulated, potentially reflecting their reliance on iron also. On the other side, APP prioritized core metabolism and downregulated non-essential pathways, such as carbon source transport (*man* operon), catabolism (*fdo* operon and *lld* operon), glycogen synthesis (*glg* operon), amino acid utilization (*sda* operon), and the synthesis of iron-containing enzymes (such as Fe-S proteins) to promote survival under iron starvation (Klitgaard et al., 2010).

#### 3.4.3 Classical iron-responsive elements

Due to the ferroxidase Dps of *Escherichia coli* that can protect bacteria from reactive oxygen species damage, the Dps-like protein FtpA in APP was also identified to possess a conserved ferritin domain containing a ferroxidase site, which plays critical roles in antioxidative stress and virulence (Tang et al., 2022). The *ftpA* gene (APPSER1_08165) was downregulated under iron-restricted conditions at the transcript level. In addition, we also saw that multiple ribosomal proteins—L33, L34, L29, S20, L24, L31, L25, S5, S16, and L27—were downregulated (>2-fold) at the protein level under iron-restricted conditions. As the classical iron-responsive elements, the roles of ribosomal proteins in iron homeostasis of APP need to be further investigated.

#### 3.4.4 Other downregulated genes

Comparison of transcriptomic and proteomic profiles showed that the downregulated DEPs generally followed the same trend as the downregulated DEGs. However, the magnitude of downregulation for proteins such as *afu* operon, *macA*, *ompW*, and *lamB* was less pronounced at the protein level than at the transcript level. Both *afuABC* and *macA* genes were regulated by *fur* and exhibited transcriptional upregulation under iron deficiency (Hsu et al., 2003). However, in this study, the transcription of *afuA*, *afuC*, and *macA* was significantly downregulated. This observation may reflect an adaptive strategy under extreme iron starvation by downregulating these genes to conserve ATP while switching to low-energy iron acquisition systems.

### 3.5 Upregulated genes under iron starvation

**Figure 5:**
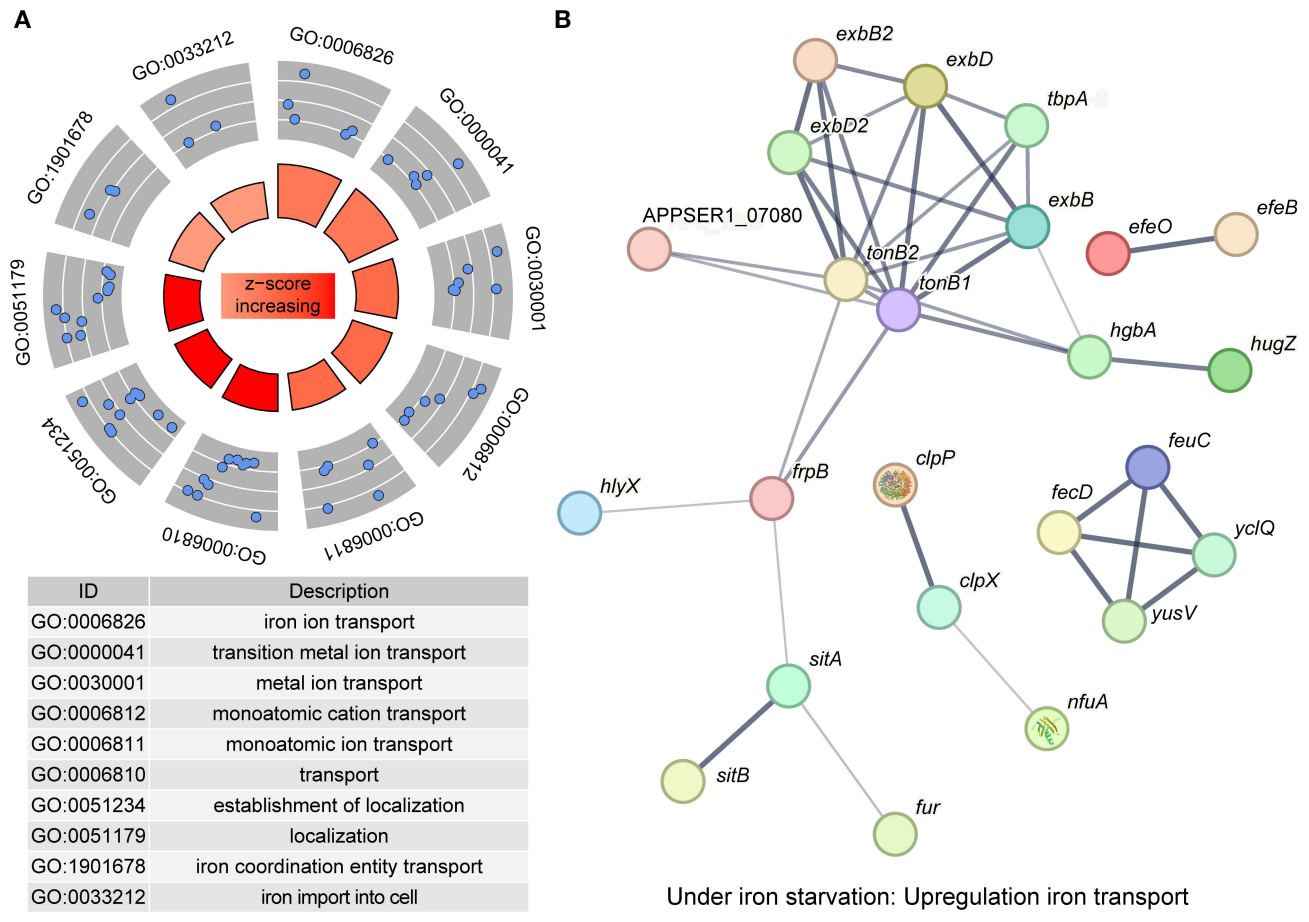
Upregulated genes in APP under iron starvation. **(A)** Enrichment of upregulated genes based on the GO database. Each blue dot represented a protein enriched in the GO term. The *z*-score in the inner circle was used to estimate the degree to which the pathway may be activated. **(B)** A protein interaction network was constructed by protein-protein interaction information from the STRING database. Each node in the network represents a distinct protein, and the thickness of the edges connecting the nodes indicates the strength of evidence for the protein-protein interaction.

**Figure 6:**
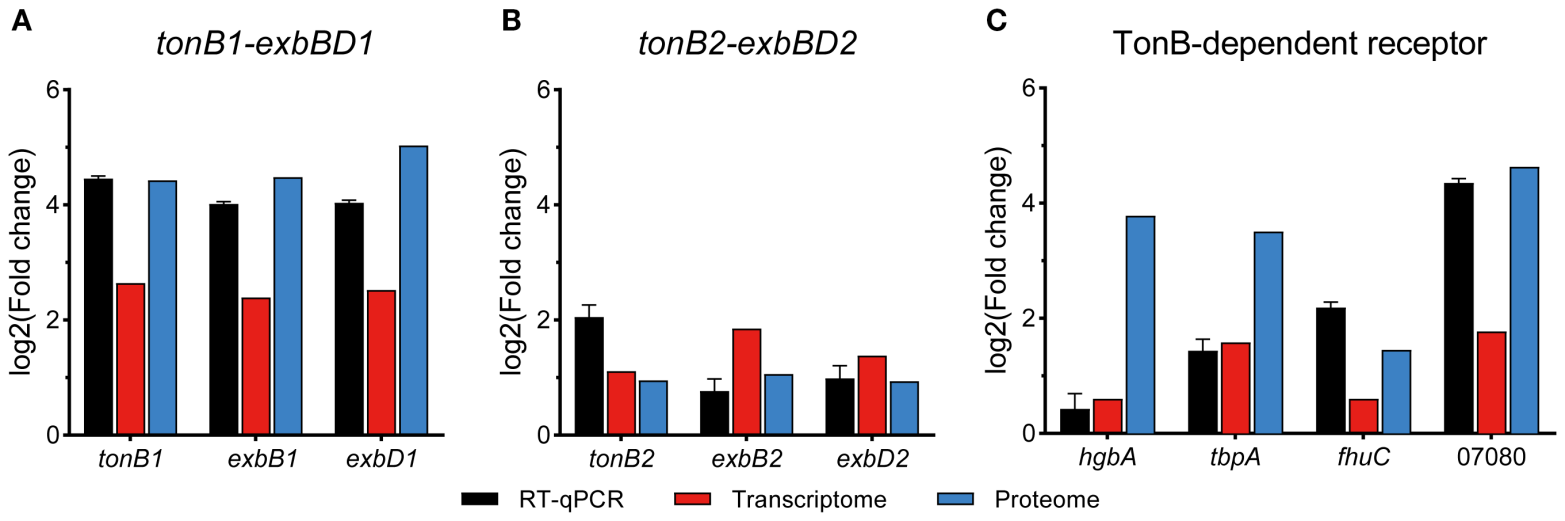
qRT-PCR verification of relative expression levels of iron utilization genes in APP under iron starvation.

In addition to downregulating the bypass metabolic pathways, APP also actively enhanced iron transport to cope with iron starvation. We screened all DEGs (458) and DEPs (532) to identify significantly upregulated genes/proteins associated with iron acquisition. Functional enrichment analysis of these upregulated entities revealed predominant association with iron transport (11/30, 36.67%) and iron binding (9/30, 30%) (**Figure 5A**). The expression profiles of key genes involved in iron acquisition, energy transduction, iron transport, transcriptional regulation, and stress resistance are detailed in **Table 2**. The interaction network among these pathways is shown in **Figure 5B**. The qRT-PCR results further confirmed the upregulation of *tonB-exbBD* systems and some specific TonB-dependent receptors, which were consistent with transcriptomic and proteomic analysis (**Figure 6**). The main upregulated gene clusters in APP upon iron-restricted conditions are listed below.

**Table 2:** Upregulated genes in the transcriptome and proteome of APP under iron starvation.

Function	Gene operon	Gene	Accession ID^a^	Transcriptome		Proteome		Description
				Log2FC^b^	*P*-value	Log2FC	*P*-value	
TonB-exbBD system	*tonB1-exbB1-exbD1*	*tonB1*	APPSER1_08640	2.64	5.90E−14	4.43	2.80E−05	Energy transducer TonB
		*exbB1*	APPSER1_08635	2.39	6.48E−25	4.48	9.33E−06	MotA/TolQ/ExbB proton channel family protein
		*exbD1*	APPSER1_08630	2.52	7.27E−27	5.03	9.72E−05	Biopolymer transporter ExbD
	*tonB2-exbB2-exbD2*	*tonB2*	APPSER1_00405	1.11	5.26E−05	0.95	8.01E−02	Energy transducer TonB
		*exbD2*	APPSER1_00410	1.38	1.59E−05	0.94	2.98E−02	TonB system transport protein ExbD
		*exbB2*	APPSER1_00415	1.85	1.23E−15	1.06	1.91E−02	TonB-system energizer ExbB
Transferrin utilization system	*tbp*	*tbpA*	APPSER1_08620	1.58	1.60E−10	3.51	4.80E−05	Lactoferrin/transferrin family TonB-dependent receptor
		*tbpB*	APPSER1_08625	1.80	1.22E−15	3.19	2.88E−04	Transferrin-binding protein-like solute binding protein
Ferrichrome utilization system	*fhu*	*fhuC*	APPSER1_10940	0.60	1.84E−02	1.45	2.16E−02	Fe^3+^-hydroxamate ABC transporter ATP-binding protein FhuC
		*fhuD*	APPSER1_10945	0.80	1.31E−03	0.93	2.70E−02	Iron-siderophore ABC transporter substrate-binding protein
		*fhuB*	APPSER1_10950	0.25	2.46E−01	–^c^	–	Fe(3+)-hydroxamate ABC transporter permease FhuB
		*fhuA*	APPSER1_10955	-0.39	4.45E−02	–	–	TonB-dependent siderophore receptor
	*fec*	*fecA*	APPSER1_03805	0.36	3.55E−02	1.80	2.30E−03	Enterochelin ABC transporter substrate-binding protein
		*fecB*	APPSER1_03810	1.84	1.24E−38	1.24	1.00E−01	Iron ABC transporter permease
		*fecC*	APPSER1_03815	1.04	7.61E−07	1.06	6.93E−02	Iron ABC transporter permease
		*fecD*	APPSER1_03820	0.99	1.28E−06	1.17	1.43E−02	Iron ABC transporter ATP-binding protein
	*cir*	*cirA*	APPSER1_05110	0.21	2.66E−01	−0.33	1.30E−01	TonB-dependent receptor
Hemoglobin/heme utilization system	*hgb*	*hgbA*	APPSER1_05725	0.60	3.74E−03	3.78	1.54E−03	TonB-dependent hemoglobin/transferrin/lactoferrin family receptor
		*hutZ*	APPSER1_05730	0.99	6.76E−07	3.51	1.03E−03	Heme utilization protein HutZ
Transcription regulator	*fnr*	*hlyX/fnr*	APPSER1_03520	2.90	1.25E−39	0.68	3.30E−02	Transcriptional regulator FNR
	*fur*	*fur*	APPSER1_06640	0.75	1.52E−04	–	–	Ferric iron uptake transcriptional regulator
Efflux pump systems		*copA*	APPSER1_06865	−0.86	2.10E−06	1.47	8.01E−03	Cadmium-translocating P-type ATPase
	*Acr*	*acrA*	APPSER1_03195	−0.05	7.66E−01	0.70	2.55E−03	Efflux RND transporter periplasmic adaptor subunit
		*acrB*	APPSER1_03200	−0.12	3.99E−01	0.77	9.23E−04	Transporter
	*sap*	*sapC*	APPSER1_04235	0.30	5.76E−02	0.83	1.97E−03	Antimicrobial peptide ABC transporter permease SapC
		*sapB*	APPSER1_04240	0.26	1.19E−01	0.64	1.86E−01	Antimicrobial peptide ABC transporter permease SapB
		*sapA*	APPSER1_04245	−0.08	6.39E−01	0.46	6.43E−03	ABC transporter substrate-binding protein
Stress resistance		*nfuA*	APPSER1_00780	1.39	6.75E−11	0.47	1.49E−01	Iron-sulfur cluster biogenesis protein NfuA
	*sit*	*sitB*	APPSER1_01425	1.13	9.76E−08	1.86	2.87E−04	Manganese transporter
		*sitA*	APPSER1_01430	0.65	4.08E−04	3.16	1.23E−03	Metal ABC transporter substrate-binding protein
	*efe*	*efeO*	APPSER1_03570	0.18	6.20E−01	3.27	6.81E−03	EfeM/EfeO family lipoprotein
		*efeB*	APPSER1_03575	0.46	1.33E−02	3.42	2.76E−03	Deferrochelatase/peroxidase EfeB
		*——*	APPSER1_03580	0.15	3.70E−01	3.37	2.85E−03	Fe^2+^/Pb^2+^ permease
Unknown		*——*	APPSER1_01450	−0.01	9.63E−01	1.45	5.58E−03	TonB-dependent receptor
		*——*	APPSER1_07080	1.77	3.59E−11	4.63	5.90E−06	TonB-dependent receptor
		*yclQ*	APPSER1_03805	0.36	3.55E−02	1.80	2.30E−03	Enterochelin ABC transporter substrate-binding protein
		*fecD*	APPSER1_03810	1.84	1.24E−38	1.24	1.00E−01	Iron ABC transporter permease
		*feuC*	APPSER1_03815	1.04	7.61E−07	1.06	6.93E−02	Iron ABC transporter permease
		*yusV*	APPSER1_03820	0.99	1.28E−06	1.17	1.43E−02	Iron ABC transporter ATP-binding protein
		*gntP*	APPSER1_00740	−0.42	1.47E−01	4.58	1.97E−03	GntP family permease
		*glxK*	APPSER1_00745	−0.40	1.67E−01	4.34	1.77E−03	Glycerate kinase
		*lldD*	APPSER1_10140	0.76	2.97E−01	3.38	2.89E−05	Alpha-hydroxy-acid oxidizing enzyme
		*chuW*	APPSER1_08325	−0.18	4.91E−01	2.23	4.28E−05	Putative heme utilization radical SAM enzyme HutW
		*——*	APPSER1_09785	−1.64	7.26E−06	2.98	1.54E−04	Iron ABC transporter substrate-binding protein
		——	APPSER1_09790	−0.88	3.75E−05	2.93	5.18E−04	Iron ABC transporter permease
		——	APPSER1_09795	−1.39	1.17E−08	1.49	1.90E−02	ABC transporter ATP-binding protein
		——	APPSER1_09800	−1.85	8.36E−15	2.38	1.63E−03	Pseudoazurin
		——	APPSER1_09805	−1.38	3.58E−13	2.38	1.91E−04	Iron ABC transporter substrate-binding protein
		——	APPSER1_09810	−0.14	4.94E−01	0.91	1.84E−03	Glutathione synthetase

a Accession ID in the genome of APP serovar 1 reference strain 4074 (CP029003.1).

b Mean of three independent samples; FC, fold change.

c No available data.

#### 3.5.1 TonB-ExbBD energy transduction systems

APP has two TonB-ExbBD systems, which play an essential role in iron homeostasis (Beddek et al., 2004). Previous studies showed that both systems were upregulated upon iron starvation, while the upregulation of *tonB2* was lower than in *tonB1* (Deslandes et al., 2007; Klitgaard et al., 2010). In this study, integrative transcriptomic and proteomic analysis revealed similar results (**Table 2**, **Figure 6**), and the TonB1-ExbB1-ExbD1 system exhibited significant upregulation with 6.25- and 21.59-fold increase of TonB1 at both the transcript and protein levels, respectively. In contrast, the TonB2-ExbB2-ExbD2 system showed moderate upregulation with 2.16- and 1.93-fold increase of TonB2, correspondingly. Collectively, these results confirm that APP significantly activated the TonB-ExbBD energy transduction systems under iron starvation.

#### 3.5.2 Iron uptake from host sources

APP can use host iron derived from hemoglobin or transferrin for growth in a process mediated by surface receptor proteins HgbA or TbpA/B, respectively (Baltes et al., 2002; Jacques, 2004; Srikumar et al., 2004). In addition to the similar transcriptomic upregulation (Deslandes et al., 2007; Klitgaard et al., 2010), proteomic analysis also showed that APP significantly upregulated transferrin receptors (11.42- and 9.13-fold for *tbpA* and *tbpB*) and the hemoglobin/heme system (13.75- and 11.41-fold for *hgbA* and *hutZ*) to compete for host iron sources (**Table 2**). Notably, an uncharacterized TonB-dependent receptor APPSER1_07080, which was designated as a putative TonB-dependent haem receptor (APL_1299; Klitgaard et al., 2010), exhibited dramatic upregulation with 3.40- and 24.77-fold at both the transcript and protein levels, respectively, potentially recognizing a novel or unknown iron transporter. The tellurite resistance gene *tehB*, which was predicted to be involved in haem utilization (Whitby et al., 2010; Klitgaard et al., 2010), was also upregulated 2.5-fold at the protein level. A putative *hpuB* gene cluster APPSER1_10635 to APPSER1_10645 involved in hemoglobin transport, corresponding to APL_1953 to APL_1955 in Klitgaard et al. (2010), had not been detected or showed no significant difference at both the transcript and protein levels, which was inconsistent with previous reports (Deslandes et al., 2007; Klitgaard et al., 2010). However, the adjacent genes APPSER1_10650 and APPSER1_10655 were upregulated at the transcript level, similar to previous reports (Deslandes et al., 2007; Klitgaard et al., 2010).

#### 3.5.3 Iron uptake by siderophores

Siderophore-mediated iron acquisition plays an important role in bacterial growth and fitness under an iron-limited environment (Ellermann and Arthur, 2017). Currently, ferrichrome is the only identified ferric hydroxamate siderophore used by APP. The transcriptomic and proteomic analysis revealed no significant difference in the *fhu* operon except the modest increase of *fhuC* (2.74-fold at the protein level) and further confirmed that the ferrichrome receptor *fhuA* is not regulated by iron (Mikael et al., 2003; Deslandes et al., 2007; Klitgaard et al., 2010). Notably, APP was able to use exogenous catecholate siderophore such as enterobactin for growth (Diarra et al., 1996). It was predicted that the *fec*-like operon and the hypothetical *cirA* protein may encode the catecholate receptors (Klitgaard et al., 2010). The *fec*-like operon APPSER1_03805 to APPSER1_03820 was significantly upregulated at both transcript and protein levels, corresponding to the CeuBCDE system in *Campylobacter* enterobactin utilization (Miller et al., 2009). However, the *cirA* gene (APPSER1_05110) showed no significant difference. The catecholate-mediated iron uptake system in APP needs to be further investigated.

#### 3.5.4 Transcriptional regulators

The iron-responsive transcriptional regulators HlyX (FNR) and Fur were involved in iron homeostasis in APP (Jacobsen et al., 2005; Buettner et al., 2009). The *hlyX* gene (APPSER1_03520) was significantly upregulated at both the transcript and protein levels, likely coordinating the repression of iron-consuming processes (e.g., anaerobic metabolism) and potentially synergizing with other iron starvation responses (Buettner et al., 2009). Fur is recognized as a key positive regulator of APP virulence (Jacobsen et al., 2005), acting primarily as a repressor of virulence genes under iron-replete conditions, and this repression is alleviated during iron starvation (Stoffel and Drakesmith, 2024). In this study, no significant difference in *fur* expression was observed, suggesting that iron deficiency may directly relieve *fur*-mediated repression without necessitating increased Fur protein abundance.

#### 3.5.5 Efflux pump systems

To counteract the metal stress, bacteria have evolved a range of efflux pump systems, including the heavy metal efflux family, the P-type ATPase family, and the cation diffusion facilitator family (Sharma et al., 2023). The P-type ATPase CopA (APPSER1_06865) in APP contributing to copper resistance was significantly upregulated at the protein level under iron-restricted conditions in this study (Peng et al., 2021; Klitgaard et al., 2010). Furthermore, the RND (resistance-nodulation-division) efflux pump AcrAB was widely distributed among different bacterial species and played an essential role in antimicrobial resistance (Subhadra et al., 2020). The expression of *acrA/B* (APPSER1_03195 and APPSER1_03200) was significantly upregulated at the protein level in response to iron stress. Additionally, the Sap transporter system is important for resistance to antimicrobial peptides in some Gram-negative pathogens including APP (Xie et al., 2017). The Sap operon *sapABC* (APPSER1_04235 to APPSER1_04245) was also significantly upregulated at the protein level, which may be involved in iron transport.

#### 3.5.6 Stress defense pathways

APP upregulated stress defense pathways to maintain metal homeostasis. The stress-resistant genes *nfuA* (Fe-S cluster assembly), *sit* (manganese transporter), and *efe* (low-pH Fe²^+^ uptake/heme utilization) were all upregulated (**Table 2**). For instance, the Fe-S cluster assembly/repair pathway protein NfuA was induced, presumably to maintain the function of essential iron-sulfur proteins (Lill and Freibert, 2020). The manganese uptake system (*sit* operon) was markedly upregulated, likely to combat iron starvation-induced oxidative stress (Dorman, 2023). The low-pH ferrous iron uptake and heme-derived iron utilization system (*efe* operon) was strongly elevated at the protein level, in agreement with previous reports (Deslandes et al., 2007; Klitgaard et al., 2010). The *efe* operon can enhance Fe²^+^ import and heme-derived iron utilization, potentially contributing to peroxidase activity (Grosse et al., 2006).

#### 3.5.7 Other upregulated genes

We found that the GntP family permease APPSER1_00740 and the glycerate kinase APPSER1_00745 dramatically increased the expression at the protein level (23.9- and 20.2-fold, respectively), which were involved in gluconate uptake and gluconate phosphorylation (Qu et al., 2025). In agreement with previous reports (Deslandes et al., 2007; Klitgaard et al., 2010), the genes encoding L-lactate dehydrogenase LldD (APPSER1_10140) and a putative heme utilization radical SAM enzyme ChuW (APPSER1_08325) were both significantly upregulated at the protein level. An operon (APPSER1_09785 to APPSER1_09810), encoding putative iron transport-associated proteins, was also dramatically upregulated at the protein level. Notably, the gene encoding ribosome-bound ATPase RbbA (APPSER1_04465) in *E. coli*, specifically bound to 70S ribosomes and 30S subunits (Kiel and Ganoza, 2001), showed the highest upregulation at the protein level (693.5-fold). These elevated expressions may represent a compensatory response of APP to accelerate iron acquisition, which also suggested to be the major virulence factors of APP (Soto Perezchica et al., 2023).

### 3.6 Exploring potential vaccine candidates in APP

**Figure 7:**
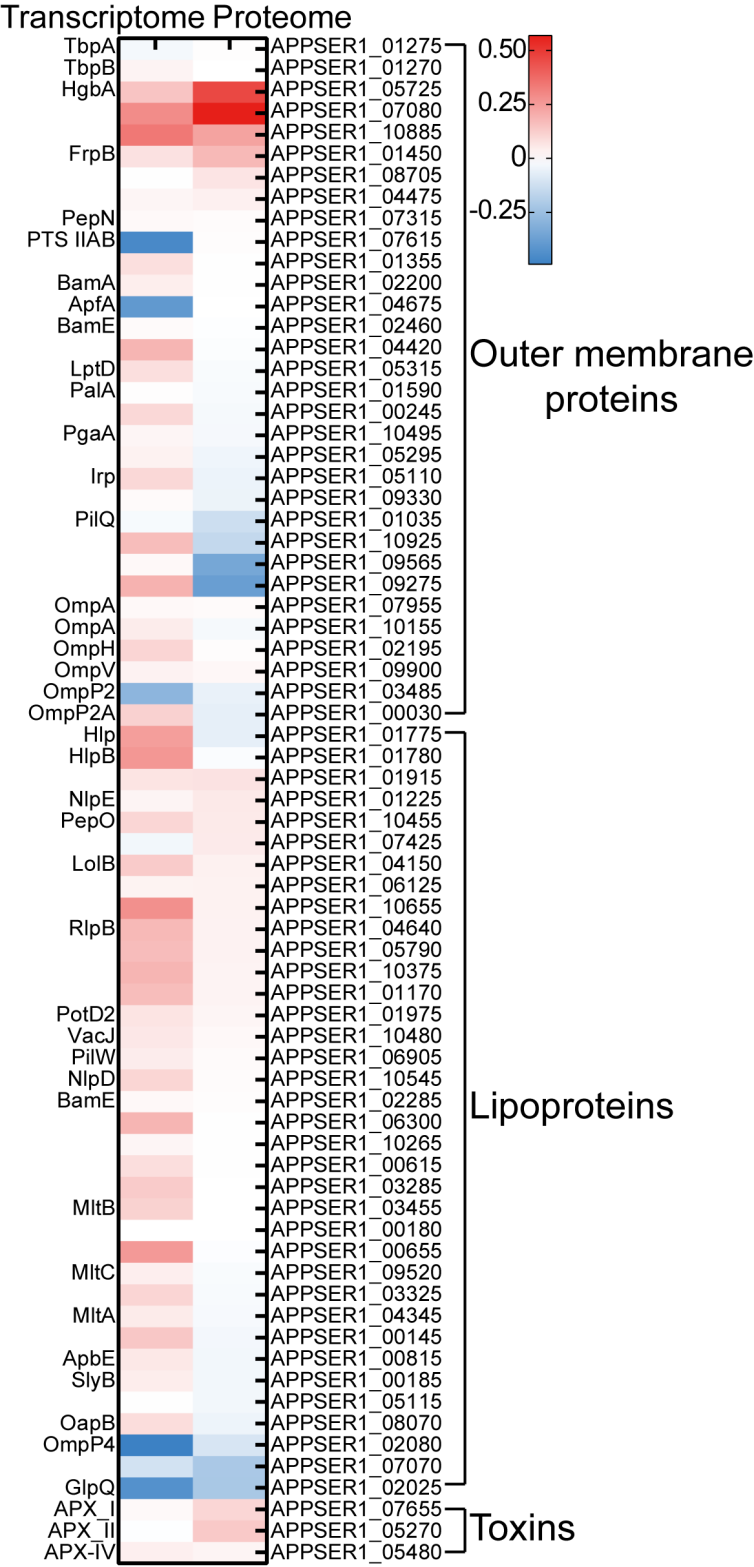
Heatmap of the expression of potential vaccine candidates under iron starvation in transcriptomic and proteomic sequencing. The scale bar represented the logarithm of normalized relative expression levels.

Currently, commercially available vaccines of APP, including inactivated bacterins and subunit vaccines, have been licensed for use in pigs with clinical limitations such as side effects and low cross-protection (Zhang et al., 2022). Innovative vaccine development depends on exploring novel and effective antigen candidates in bacteria (Loera-Muro and Angulo, 2018). Iron transporters and iron-regulated proteins, being major virulence factors, represent promising targets for vaccine development (Goethe et al., 2000; Stoffel and Drakesmith, 2024). For instance, the commercial subunit vaccines of APP include the iron-regulated proteins such as TbpA/B and OmlA (Loera-Muro and Angulo, 2018). Multiple iron transporters and iron-regulated proteins, including TbpA/B, OmlA, HgbA, LppC, and LolB, had been evaluated for the APP subunit vaccine (Loera-Muro and Angulo, 2018). The detergent extraction of APP cultures induced by iron restriction was also used for a subunit vaccine strategy (Goethe et al., 2000). The 2-D immunoblot-based proteomic and immunoproteomic analysis was used for exploring the potential APP subunit vaccines under iron-restricted conditions (Chung et al., 2012; Buettner et al., 2011). In this study, integrated transcriptomic and proteomic analysis revealed that iron starvation induced significant expression of a range of outer membrane proteins, lipoproteins, and Apx toxins (**Figure 7**); among them, several TonB-dependent receptors with undefined functions, such as APPSER1_07080 and APPSER1_10885, can be used for further investigation on subunit vaccine development.

## 4 Conclusion

This study aims to further analyze the adaptive mechanisms of APP under iron-restricted conditions. We seek to elucidate the mechanisms enabling APP to enhance growth and colonization within the host while maintaining iron homeostasis, utilizing transcriptomics to investigate regulatory responses and proteomics to validate functional protein expression. We observed significant changes at both the transcriptional and protein levels in the core metabolic cycles of APP. Simultaneously, under iron starvation conditions, APP activated multiple iron acquisition systems to enable efficient exploitation of host-associated iron, siderophores, and free Fe²^+^. In conclusion, integrative transcriptomic and proteomic profiling of APP under iron starvation provides a comprehensive understanding of the mechanisms underlying resource conservation and adaptation to iron stress in APP. Furthermore, the results highlight novel potential targets for developing vaccines against APP.

## Data Availability

The datasets presented in this study can be found in online repositories. The raw data of transcriptomic sequencing are deposited in the NCBI database under the BioProject number PRJNA1284040. The proteomic data are deposited in the iProX database under the Project ID IPX0012451001.

## Supplementary material

Supplementary Figure 1 Growth curves of APP under different culture conditions. (A) Growth curves of APP in the TSB medium with addition of different concentrations of DFO. (B) Growth curves of APP in the TSB medium with or without addition of FeCl_3_.

Supplementary Figure 2 Metabolism pathway analysis between proteins in the cluster of shared DEGs/DEPs with the cluster of DEPs. The red line represented the pathways in the cluster of shared DEGs/DEPs, the green line represented the pathways affected by the cluster of DEPs. The metabolism pathway analysis was performed by iPath3.0 (https://pathways.embl.de/).
