# Supplementary figures and images for: Transcriptomic and proteomic profiling of Actinobacillus pleuropneumoniae responses to iron starvation
Source: Front Cell Infect Microbiol. 2025 Sep 25;15:1669654. doi: 10.3389/fcimb.2025.1669654 (PMC12507848; doi:10.3389/fcimb.2025.1669654)

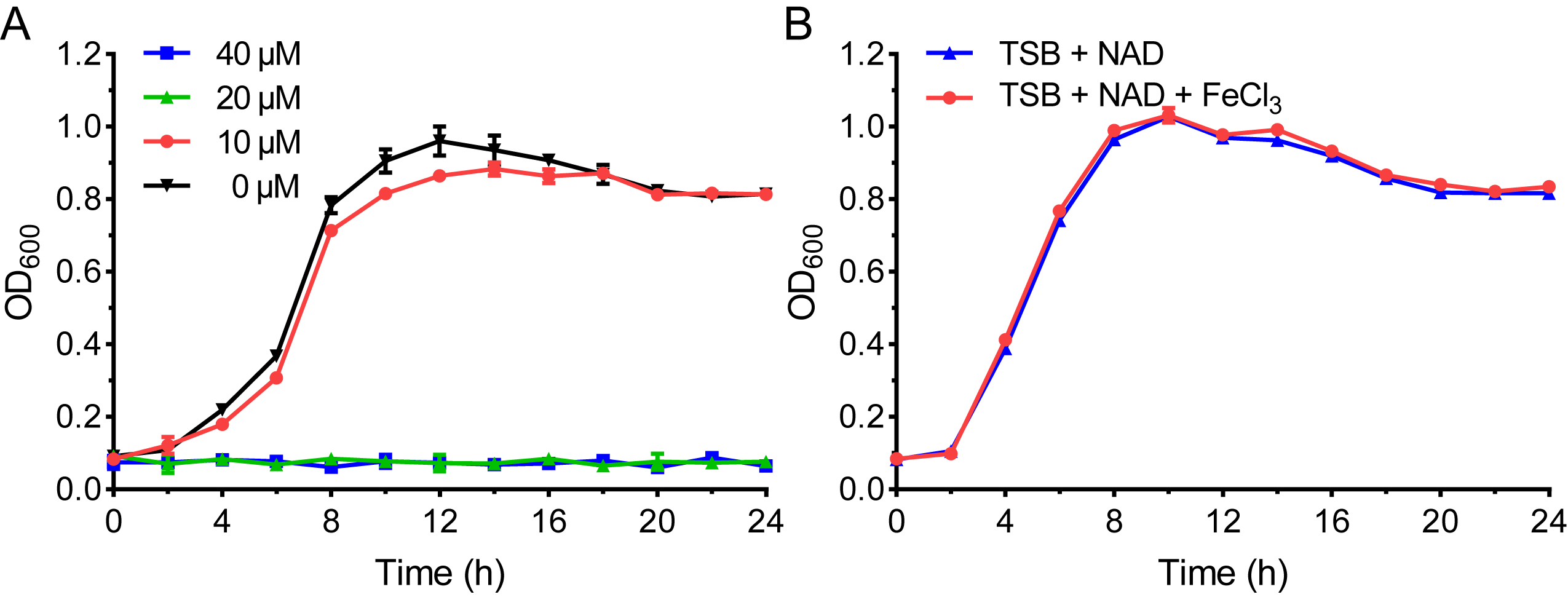

Supplement: Supplementary Figure 1 — Growth curves of APP under different culture conditions. (A) Growth curves of APP in the TSB medium with addition of different concentrations of DFO. (B) Growth curves of APP in the TSB medium with or without addition of FeCl3. [file Image1.tif]
